# *Lens orientalis* Contributes Quantitative Trait Loci and Candidate Genes Associated With Ascochyta Blight Resistance in Lentil

**DOI:** 10.3389/fpls.2021.703283

**Published:** 2021-08-26

**Authors:** Rama Harinath Reddy Dadu, Ido Bar, Rebecca Ford, Prabhakaran Sambasivam, Janine Croser, Federico Ribalta, Sukhjiwan Kaur, Shimna Sudheesh, Dorin Gupta

**Affiliations:** ^1^School of Agriculture and Food, Faculty of Veterinary and Agriculture Sciences, Dookie College, The University of Melbourne, Dookie, VIC, Australia; ^2^Grains Innovation Park, Agriculture Victoria, DJPR, Horsham, VIC, Australia; ^3^Centre for Planetary Health and Food Security, Griffith University, Nathan, QLD, Australia; ^4^Centre for Plant Genetics and Breeding, School of Agriculture and Environment, The University of Western Australia, Crawley, WA, Australia; ^5^Agriculture Victoria, AgriBio, Centre for Agri Bioscience, Bundoora, VIC, Australia

**Keywords:** lentil, *Lens orientalis*, Ascochyta blight, genetic linkage map, quantitative trait loci, candidate genes

## Abstract

Australian lentil production is affected by several major biotic constraints including Ascochyta blight (AB), caused by *Ascochyta lentis,* a devastating fungal disease. Cultivation of AB resistant cultivars, alongside agronomic management including fungicide application, is the current most economically viable control strategy. However, the breakdown of AB resistance in cultivars, such as Northfield and Nipper, suggests the need for introgression of new and diverse resistance genes. Successful introgression entails an understanding of the genetic basis of resistance. In this context, a biparental mapping population derived from a cross between a recently identified AB resistant accession ILWL 180 (*Lens orientalis*) and a susceptible cultivar ILL 6002 was produced. A genetic linkage map was constructed from single-nucleotide polymorphism markers generated using a genotyping-by-sequencing transcript approach. Genetic dissection of the mapping population revealed a major quantitative trait loci (QTL) region nested with three QTLs on linkage group 5 and explained 9.5–11.5 percent (%) of phenotypic variance for AB resistance. Another QTL was identified on LG2 with phenotypic variance of 9.6%. The identified QTL regions harbored putative candidate genes potentially associated with defense responses to *A. lentis* infection. The QTL analysis and the candidate gene information are expected to contribute to the development of diagnostic markers and enable marker-assisted resistance selection in lentil breeding programmes.

## Introduction

Lentil (*Lens culinaris* Medikus), a member of the Fabaceae legume family, is cultivated across the world for its high dietary benefits. Owing to the demand and export value, Australian lentil production scaled to 255,185 MT in 2018, an increase of over 85% compared to 3,000 MT produced in 1990 (Faostat, 2021). However, lentil productivity is highly inconsistent and ranged between 0.8 and 2.4tonnes/ha in the last decade (Faostat, 2021). A major constraint to lentil productivity is yield reduction resulting from infection by the fungal disease ascochyta blight (AB) caused by *Ascochyta lentis*, estimated to cost the Australian lentil industry 16.2 million AUD per year (Murray and Brennan, 2012). Globally, AB is considered the most widespread and economically important disease in lentil crops (Erskine et al., 1993; Ye et al., 2002). Yield losses of up to 70% are common where conditions favorable for *A. lentis* spread frequently occur (Gossen and Morrall, 1983; Kaiser, 1992). Use of resistant cultivars is considered an effective control measure in combination with appropriate cultural methods and application of fungicides (Hawthorne et al., 2015). In Australia, prominent variety releases, such as PBA Northfield, Nipper, PBA Jumbo2 and PBA Hurricane XT with improved AB resistance (Rodda et al., 2017; Pulse Australia, 2020), gave hope of a genetic solution to this problem. However, the emergence of highly aggressive *A. lentis* isolates has contributed to the breakdown of AB resistance of lentil cultivars with similar genetic background, particularly those that derive resistance from cv. Northfield (Davidson et al., 2016). Under controlled environment screening conditions, isolates have also been reported to have caused a susceptible reaction in cultivars (PBA Jumbo and PBA Blitz) with Indianhead pedigree (Davidson et al., 2016) and in cv. Indianhead itself (Dadu et al., 2018b). The widespread production area of cultivars with Indianhead heritage is therefore likely under current selection pressure, underlining the critical need for inclusion of novel and diverse resistance alleles/genes into the breeding programme to enhance the durability of resistance sources to AB.

Novel alleles for commercially important traits exist in the exotic germplasm of crops, including wild relatives and landraces (Wang et al., 2017). Within *Lens*, phylogenetic and structural analysis using genotyping-by-sequencing (GBS) and exome capture array by Wong et al. (2015) and Ogutcen et al. (2018) have identified four gene pools, primary (*L. culinaris/L. orientalis/L. tomentosus*), secondary (*L. lamottei/L. odomensis*), tertiary (*L. ervoides*) and quaternary gene pool (*L. nigricans*). Previous studies have revealed several useful sources of useful alleles within wild accessions of *L. orientalis*, *L. odomensis*, *L. ervoides*, *L. nigricans* and *L. lamottei* (Bayaa et al., 1994; Tullu et al., 2010). This has included alleles for seedling pigmentation, plant height, time to flower, time to maturity, biomass, number of branches, flower color, seed coat color, cotyledon color, 100 seed weight, seed yield and tolerance to abiotic stress including salinity, drought, heat and cold (Singh et al., 2018). Several sources of resistance to various diseases including AB, anthracnose, rust, wilt, stemphylium blight and the parasitic weed *Orobanche* have been reported within wild germplasm (Singh et al., 2018).

Novel allelic combinations, including those targeted at improved disease resistance, can be achieved using conventional crossing methods if, as for *Lens orientalis*, the wild relative is a subspecies or member of the same gene pool (Ladizinsky et al., 1984; Ferguson et al., 2000). To fix and select these novel allelic combinations in inbred lines following a cross, most breeding programmes commonly need six to seven generations of selfing through single-seed descent (SSD) method. In lentil, the time taken to produce six generations may vary between 2 and 4years depending on the availability of resources and is considered laborious and time consuming. Croser et al. (2016) and Ribalta et al. (2017) proposed an accelerated single-seed descent (aSSD) approach wherein rapid generation turnover was achieved through manipulation of key *in vivo* growing conditions, such as light, photoperiod and temperature and use of immature seeds. This enables production of a single generation within 56days and as many as five generations in a year thus fast-tracking cultivar development process. More recently, *L. culinaris* × *L. ervoides* recombinant inbred line (RIL) population was successfully produced in *Lens* genus using aSSD approach by Lulsdorf and Banniza (2018). Accelerated single-seed descent platforms have the capacity to speed allele fixation, gene discovery and thus genetic gain.

The usual abundance of single-nucleotide polymorphisms (SNPs) across the genomes makes them preferable markers for various downstream studies including linkage mapping, quantitative trait loci (QTL) analysis, genome wide association study (GWAS) and genomic selection (GS; Roorkiwal et al., 2020). The decreasing cost of next-generation sequencing (NGS) technologies facilitated the development of high-throughput genotyping platforms, such as widely used GBS for polymorphism detection and trait mapping (Scheben et al., 2017). GBS through transcriptomics (GBS-t) using RNA-Seq is used as an alternative approach to GBS-RAD (restriction site – associated DNA) to enable selective sequencing of regions with high proportion of functional variants and limited repetitive regions (Hirsch et al., 2014; Scheben et al., 2017). This approach has been successfully used to detect large number of SNPs in different crops including wheat (Miller et al., 2016), canola (Bancroft et al., 2011) and lentil (Malmberg et al., 2018). Polanco et al. (2019) identified genomic regions affecting time to flowering, seed size and AB resistance in lentil using SNPs obtained by transcriptome sequencing.

In lentil, SNPs contributed to the generation of high-density genetic maps and facilitated identification of QTLs linked to agronomical traits (Kumar et al., 2015). Sudheesh et al. (2016a) generated a consensus linkage map by combining three intraspecific linkage maps derived from RIL populations of Indianhead × Digger, Indianhead × Northfield and Northfield × Digger using bridging markers. Furthermore, the map allowed identification of common genomic regions between the populations conferring resistance to AB and associated to Indianhead.

However, in the wake of emergence of isolates able to overcome the existing resistances, the production of interspecific linkage maps may permit identification of novel regions to adaptive traits, such as AB resistance and widen the genetic base of current lentil breeding programmes. The candidate genes underpinning these regions may also help understand the molecular basis of defense mechanism to *A. lentis* (Malmberg et al., 2018). In this study, we aim (1) to construct high-density genetic linkage map through an GBS-t approach using RIL population developed from a cross between *L. orientalis* accession ILWL 180 (or IG 72703), highly resistant to Australian and Syrian *A. lentis* isolates and a universal susceptible *L. culinaris* cultivar ILL 6002 (Bayaa et al., 1994; Dadu et al., 2017); (2) to locate and characterize the QTLs and candidate genes associated with resistance to AB.

## Materials and Methods

### Development of Biparental Mapping Population

A segregating mapping population was developed from an interspecific cross between *L. orientalis* accession ILWL 180 and *L. culinaris* accession ILL 6002. Crosses between accessions ILWL 180 (male parent) and ILL 6002 (female parent) were made at the glasshouse at Dookie College, The University of Melbourne, Australia following the method described by Tullu et al. (2013). Out of 430 crosses made, 201 were successful. F_1_ hybrid seeds were harvested and then raised in 25cm diameter pots filled with pine bark potting mix (Australian Grow Solutions, Tyabb, VIC, Australia). Seedlings were watered on alternative days and fertilized weekly using nitrogen-enriched liquid fertilizer (Nitrosol, Amsgrow; 4.5ml/L) until maturity. One hundred ninety-eight F_2_ seeds harvested from a single F_1_ plant were used to develop the mapping population (F_2:5_). Individual F_2_ plants were grown in 15cm diameter pots at the glasshouse at Dookie College, The University of Melbourne, VIC. The F_2:5_ generations were then individually tracked and cycled under aSSD conditions modified from Croser et al. (2016) and Ribalta et al. (2017) at the Centre for Plant Genetics and Breeding, The University of Western Australia, Crawley, WA. Seeds were sown one per 0.4L pot containing pine bark: peat: sand (2.5:1:1.5) potting mix (Richgro Garden Products) at pH 7 and grown at 22°C day/18°C night, RH 60±10% and 18h photoperiod supplied by natural light supplemented and extended by Valoya AP67 Series B light emitting diode based arrays, with red:far-red ratio of 2.89 and intensity of c. 325μmol m^−2^ s^−1^ at canopy (Valoya Oy, Helsinki, Finland). Light spectra and intensity were measured using a Sekonic C7000 SpectroMaster spectrometer (Sekonic Corp., Tokyo, Japan) and calculation of spectral ratio was as per Runkle and Heins (2001). Plants were hand watered daily and fertilized weekly with N:P:K fertilizer (19:8.3:15.8) with micronutrients (Poly-feed Greenhouse Grade, Haifa Chemical Ltd. at a rate of 0.3g per pot. Flowering occurred across all lines within 30days of sowing and immature seed was removed at physiological maturity and resown to the following generation to give a generation turnover time of less than 60days. Back up seed from each line at each generation after the first to enable resowing if required. The F_4:5_ seed was left to fully mature on the plant and 5–15 seeds from 140 RILs were returned to UoM for characterization.

### Phenotypic Assessment of AB Resistance Under Controlled Environment Conditions

The disease reactions to AB were assessed within the RIL population during September 2018 using an aggressive *A. lentis* isolate FT13038, which was obtained from the South Australian Research and Development Institute (SARDI), South Australia. Isolate FT13038 was previously confirmed to effectively discriminate differential resistance responses between the accessions ILWL 180 and ILL 6002 (Dadu et al., 2017). Seeds of each RIL and parent were sown in 10cm diameter pots filled with pine bark potting mix and maintained at 18±1°C, 12h/12hday/night photoperiod, 60% RH and 300μEm^−2^ s^−1^ light intensity in a Conviron growth chamber at Dookie College, The University of Melbourne. The experiments were set out in a completely randomized design with two and three replicates of each RIL (four seeds per replication) and parent (three seeds per replication), respectively. Seedlings were watered and fertilized as mentioned above for F_1_ seedlings to promote germination and plant development. Fourteen days old seedlings were inoculated with a spore suspension prepared from isolate FT13038 as described below.

The mycelial plugs of a single spored highly aggressive isolate FT13038 received from SARDI were cultured on potato dextrose agar (PDA) media and incubated for 14days as described by Dadu et al. (2017). Spore suspension was prepared from 14days old cultures by flooding with sterile distilled water and gently disturbing the culture surface with a sterile glass rod. Conidia suspended in sterile distilled water were separated from mycelium using a 250mm pore sized sieve and the spore concentration was adjusted to 1×10^6^ spores/ml using a hemocytometer. The spore suspension was then supplemented with two to three drops of surfactant Tween 20 (0.02% v/v) and was used to inoculate RILs and parents until run off using an air pressurized hand sprayer.

The inoculated pots were covered with long inverted solid paper cups (In Hospitality, Shepparton, Australia) to maintain leaf wetness and facilitate high humidity (Chen and Muehlbauer, 2003; Dadu et al., 2017). Pots were then randomly placed in plastic crates and moved to a Conviron growth cabinet at Dookie College, The University of Melbourne. The cups were removed after 48h post-inoculation (hpi). To maintain high relative humidity and promote spore germination, the plants were misted thrice daily and plastic crates were covered with wet hessian bags until first appearance of disease symptoms. The progression of disease was assessed for each seedling at 7, 14, 21 and 28days post-inoculation (dpi; Ford et al., 1999; Sambasivam et al., 2017). The disease severity was assessed based on two disease symptoms: the leaf lesion ratio (number of infected leaves/total leaves) and stem lesion ratio (number of infected nodes/total nodes) of inoculated plants and shown as percentage diseased (%; Davidson et al., 2016; Dadu et al., 2018b). Area under disease progress curve (AUDPC) was used to measure the progression of the disease over time and was calculated as described by Campbell and Madden (1990) and Dadu et al. (2017). The disease severity data of parents and RILs were transformed using square-root and confirmed to be normally distributed using Shapiro–Wilk test for normality. The data were further analyzed for differences between the genotype groups (RIL and parents) using analysis of variance (ANOVA) in Genstat^®^ version 16.1.0.10916 (64-bit edition, VSN International Limited, United Kingdom). Differences between the parents at each time point (7, 14, 21 and 28 dpi) were assessed based on Tukey’s least significant difference (LSD) at 95% confidence interval.

### RNA Extraction, cDNA Library Construction and Illumina Sequencing

Total RNA was extracted from selected young leaves of 14–21day old seedlings of parents and 140 RILs using the RNeasy^®^ 96 kit (Qiagen Inc., Hilden, Germany) following the manufacturer’s instructions. The integrity and purity of the total RNA were determined with a TapeStation 2200 platform (Agilent Technologies, Santa Clara, CA, United States) following the manufacturer’s instructions. Total RNA concentration and purity were further confirmed using a NanoDrop 2000 (Thermo-Scientific, Wilmington, DE, United States) at two wavelength ratios of A260/230 and A260/280nm. RNA-Seq cDNA libraries were prepared using a SureSelect Strand-Specific mRNA Library Preparation kit (Agilent Technologies, Santa Clara, CA, United States). This entailed isolation of poly(A) RNA from total RNA, fragmentation of poly(A) RNA, synthesis of double-stranded cDNA, adapter-ligation and indexing of cDNA libraries. The quality of the libraries was assessed using a TapeStation 2200 platform with a D1000 Screen Tape System and equal quantities of each cDNA library with a unique barcode were then pooled to create a single pooled sample suitable for NGS. The multiplexed sample was quantified with a KAPA library qPCR quantification kit (KAPA Biosystems, Boston, MA, United States) and sequenced using a HiSeq 3000 system (Illumina Inc., San Diego, CA, United States).

### Variant Calling and Filtering of SNP

The sequence output was demultiplexed and parsed into individual libraries according to the barcodes. Following fastq data generation, the raw sequence reads were filtered using a custom perl script and Cutadapt v1.4.1 (Martin, 2011) software. Reads were filtered by removing adaptor sequences along with reads and bases of low quality (discarding reads which have more than 10% bases with Q ≤20). Reads which had three consecutive N’s and >3 consecutive nucleotides with Phred scores ≤20 were trimmed. Finally, any reads that were shorter than 50bp were removed from the final set. The cleaned and trimmed reads were then mapped to the cultivar Cassab reference transcriptome (Sudheesh et al., 2016b) using Burrows Wheeler Aligner (BWA) v0.7.17 with the Mem algorithm (Li, 2013). Firstly, SNPs between the RIL parents were recorded in the sequence alignment and mapping (SAM) format. The SAM files were converted into binary format (BAM) files, sorted and indexed using SAMtools (version 1.9) sort and view options. BAM files were processed for variant calling through SAMtools mpileup and bcftools (version 1.9) and filtered using vcftools (v0.1.16; Danecek et al., 2011). Variant calling in RILs was then performed using a SNP list (biallelic, homozygote in each parent and polymorphic between parents) generated from the parents. Variants with minimum allele depth of 10, average mapping quality score above 30 and minor allele frequency larger than 0.05 were retained. Markers differing from the above criteria were converted into missing values and those with >10% missing data were subsequently removed.

### Genetic Linkage Map Construction

Prior to the construction of a high-quality linkage map, the heterozygote calls were reset to ‘./.’ and were subsequently considered as missing. Markers and genotypes were verified for excessive segregation distortion (5% significance level), genotypes with a high proportion of matching alleles (>95%) and high rates of missing calls (>20%). An additional pre-mapping filtering step was applied to remove any non-polymorphic (between the parents) and multi-allelic loci (number of alleles >2). The SNPs were further phased to ensure consistency of the alleles that were contributed from the male or female parent using a custom script in the R statistical computing environment (R Development Core Team, 2015). Finally, missing call rates were recalculated across markers and genotypes to retain only markers and genotypes with missing rates ≤0.3. The filtered, high-quality SNPs were then clustered into linkage groups (LG) to construct a high-density linkage map using the ‘mstmap’ function from within the ASMap R package (v1.0-4; Taylor and Butler, 2017). The ‘mstmap’ function was invoked with the following parameters: ‘Kosambi’ distance calculating function (dist.fun), missing threshold (miss.thresh) of 10% and population type (pop.type) RIL5 and value of *p* (p.value) of 5.12e^−12^. Unlinked markers or minor LGs that comprised less than 10 markers were discarded. The generated map was further tested to identify and remove erroneous markers and genotypes showing double crossovers, highly distorted segregation patterns and missing data using the ‘profileMark’ and ‘profileGen’ functions, respectively, within the ASMap package (v1.0-4; Taylor and Butler, 2017). The map was then reconstructed and the correctness and reliability of the constructed map were confirmed by visually assessing a heatmap, produced by ASMap, showing the estimates of pairwise recombination fraction and LOD scores between each pair of markers (Taylor and Butler, 2017). The linkage map explaining the marker density within each group was plotted using the LinkageMapView R package (version 2.1.2; Ouellette et al., 2018).

### QTL Analysis and Identification of Candidate Genes

The constructed linkage map, filtered SNP data and the square-root transformed phenotypic data mentioned previously were used as input for QTL analysis using the R/qtl2 v0.12 package (Broman et al., 2018), an improved modern implementation of the original R/qtl (Broman et al., 2003). A genome scan approach was chosen to identify significant QTL regions, using a linear mixed model accounting for relationships among individuals using a random polygenic effect. This was supported by a permutation test (*n*=1,000) to determine the LOD threshold for significance for each trait at each time point. A LOD-1 confidence interval (CI) was used to identify the higher and lower limit CIs for the QTLs (LOD>3), determine the width of the QTL regions and detect the underlying loci (Snoek et al., 2018). Phenotypic variance explained by the QTL was estimated from the LOD score: 1−10^−(2/*n*)*LOD^ (Broman and Sen, 2009) at the peak position within the CI and the number of individuals assessed (*n*). Loci and QTL positions across the linkage map were prepared in R and exported in MapChart v2.32 for plotting (Voorrips, 2002).

The sequences of the loci underpinning the QTL regions were extracted from the cultivar Cassab reference transcriptome (Sudheesh et al., 2016b) and annotated using the translated Basic Local Alignment Search Tool (BLASTx) against non-redundant protein database (nr) at the National Centre for Biotechnology Information (NCBI), with a threshold of *E*-value <1e^−20^. The search results were limited to species of the Fabaceae family to reveal putative candidate genes as described by Leonforte et al. (2013). The sequences of the SNP markers flanking the QTL regions were also BLASTn (threshold *E*-value of zero) against the draft lentil genome assembly v1.2 (Bett et al., 2016) through the https://knowpulse.usask.ca/portal/blast/nucleotide/nucleotide to identify their genomic locations.

### Prediction of Candidate Gene Associated Mutations and Consequences

Coding sequences (CDS) of the putative candidate genes were predicted based on the best matching BLAST results. An in-house R script was used to determine each SNP location relative to the CDS as the 5'-untranslated region (UTR), 3'-UTR or CDS as well as the position of the SNP relative to the open reading frame (ORF) as 1, 2 or 3 for CDS. For SNPs located in the coding region, the CDS for the reference and alternative alleles was translated to their amino acid sequences and compared to determine the effect of the mutation as synonymous, non-synonymous or non-sense (introduction of a stop codon). In the case of a non-synonymous mutation, the exact position and substitution of the amino acid were recorded as AxxB, where A was the reference amino acid, xx was the amino acid position in the predicted peptide and B was the alternative amino acid, as described by Ogino et al. (2007). Sorting intolerant from tolerant (SIFT) analysis was used to predict deleterious effects to the protein function of genes with non-synonymous mutations (Kumar et al., 2009).

### Supporting Data

An online dataset containing all supporting genotyping and phenotyping data and the code required to reproduce the results, summary tables and plots found in this publication, is publicly available at Open Science Framework, DOI 10.17605/OSF.IO/T9ABQ (Dadu et al., 2021). The sequence data have been deposited at NCBI/GEO under the accession GSE176412. BioProject ID for transcriptome is PRJNA736090 while SRA ID is SRP323267.

## Results

### Response of Parents and RIL Population to AB Infection

**Figure 1:**
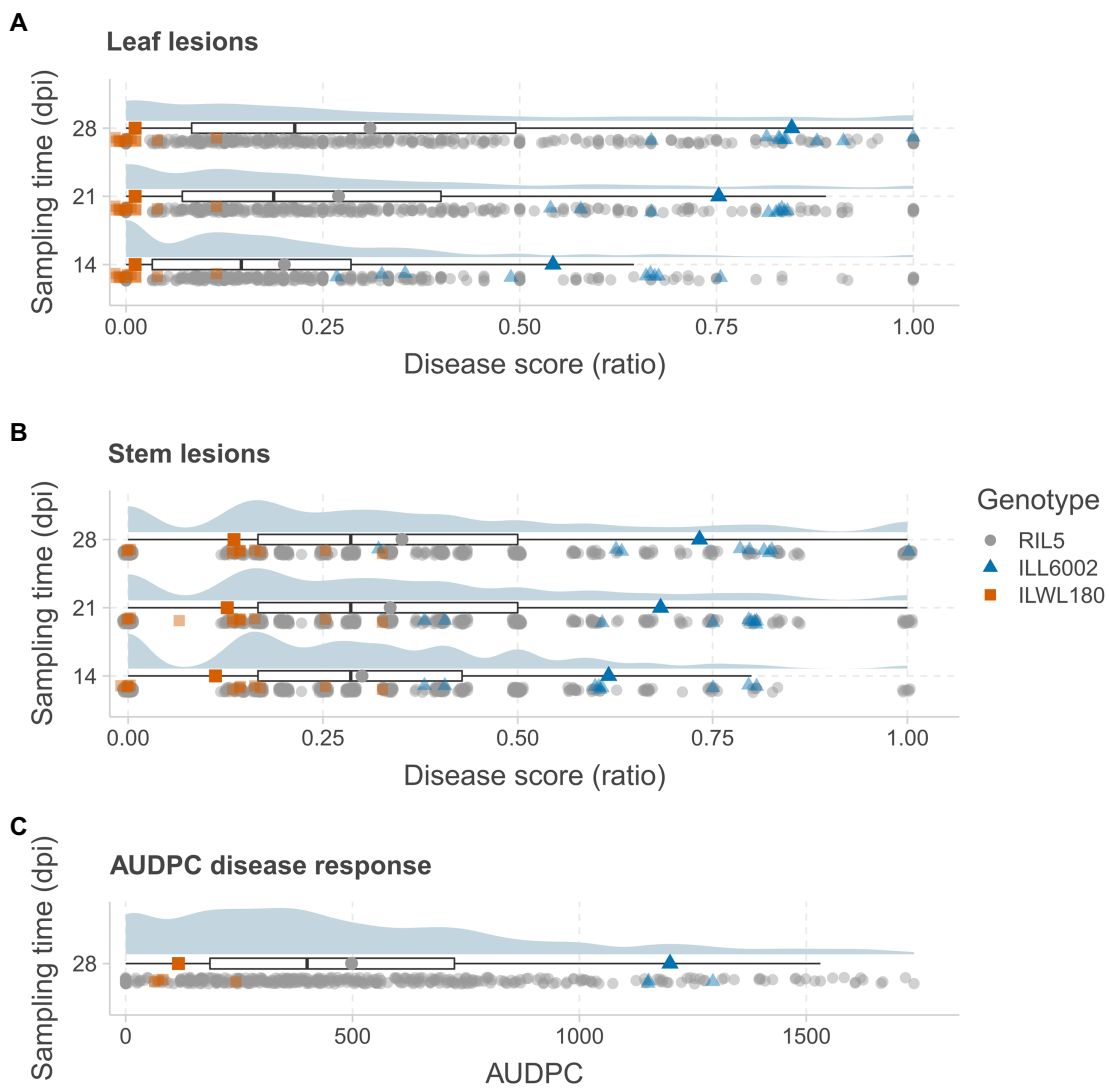
Distribution of AB disease symptoms in a RIL population derived from the cross ILWL 180 × ILL 6002. The distributions of leaf lesion scores **(A)** and stem lesion scores **(B)** are presented as ratios at 14, 21 and 28days post-inoculation (dpi) and AUDPC **(C)** at 28 dpi. The distributions are presented as raincloud plots, with the median and quantiles shown below as box plots. Each sampling data point is plotted below the box plots, with marker colors and shapes representing genotypes as per the legend (RIL – gray circles; ILL6002 – blue triangles; and ILWL 180 – orange squares). The average scores of the parents and the RIL population are presented as larger markers on the box plots.

Significant differences for AB resistance were found between the parents, ILWL 180 (Leaf lesion score at 28 dpi=0.66±0.50%, mean AUDPC=11; Stem lesion score at 28 dpi=7.77±2.21%, mean AUDPC=122) and ILL 6002 (Leaf lesion score at 28 dpi=72.66±4.37%, mean AUDPC=907; Stem lesion score at 28 dpi=63.33±4.09%, mean AUDPC=1,078) following inoculation with the highly aggressive isolate FT13038 at 14, 21 and 28 dpi (*p<*0.001; Figures 1A–C). Overall, disease severity within the RIL population ranged from 0 to 100% while AUDPC values ranged from 0 to 1750 with a mean value of 413. A high correlation between leaf and stem lesion scores (*r*=0.85) was recorded at 28 dpi. Segregation for AB resistance within the RIL population showed a monomodal and normal distribution following square-root transformation for leaf lesion ratio at 21 dpi (Shapiro–Wilk test, *W*=0.99, *p>*0.05) and 28 dpi (*W*=0.99, *p>*0.05) and stem lesion ratio at 28 dpi (*W*=0.98, *p>*0.05). This suggested that resistance to AB within the wild accession ILWL 180 was polygenic and quantitatively inherited. A small proportion of both the positive (5) and negative transgressive segregants (11) that exceeded the ranges of parental means by a minimum of one LSD value were evident in the population.

### Transcriptome Sequencing and SNP Discovery

A total of 694,694,624 paired end reads (150-bp) were generated by sequencing multiplexed cDNA libraries on the Illumina HiSeq 3000 platform with an average of 4,997,803 reads per RIL progeny. On average, 98.5% of the reads for each sample mapped to the reference transcriptome. Variant calling was performed first for the parents after alignment of uniquely mapped reads to the reference transcriptome. A total of 570,099 SNPs were initially identified from the parents and were used to call variants from the RIL data, identifying 403,423 of these SNPs in the RIL population. Sequential filtering of the identified SNPs led to the retaining of 85,748 and 6,662 SNPs from the parents and RIL population, respectively. SNPs were further phased out through additional pre-mapping filtering steps to be considered for genetic linkage mapping. Finally, loci from both parents and RILs were merged to retain only shared SNPs, resulting in 2,514 SNPs in 138 genotypes (parents and 136 RILs, Supplementary Table 1).

### Construction of a Genetic Linkage Map

**Figure 2:**
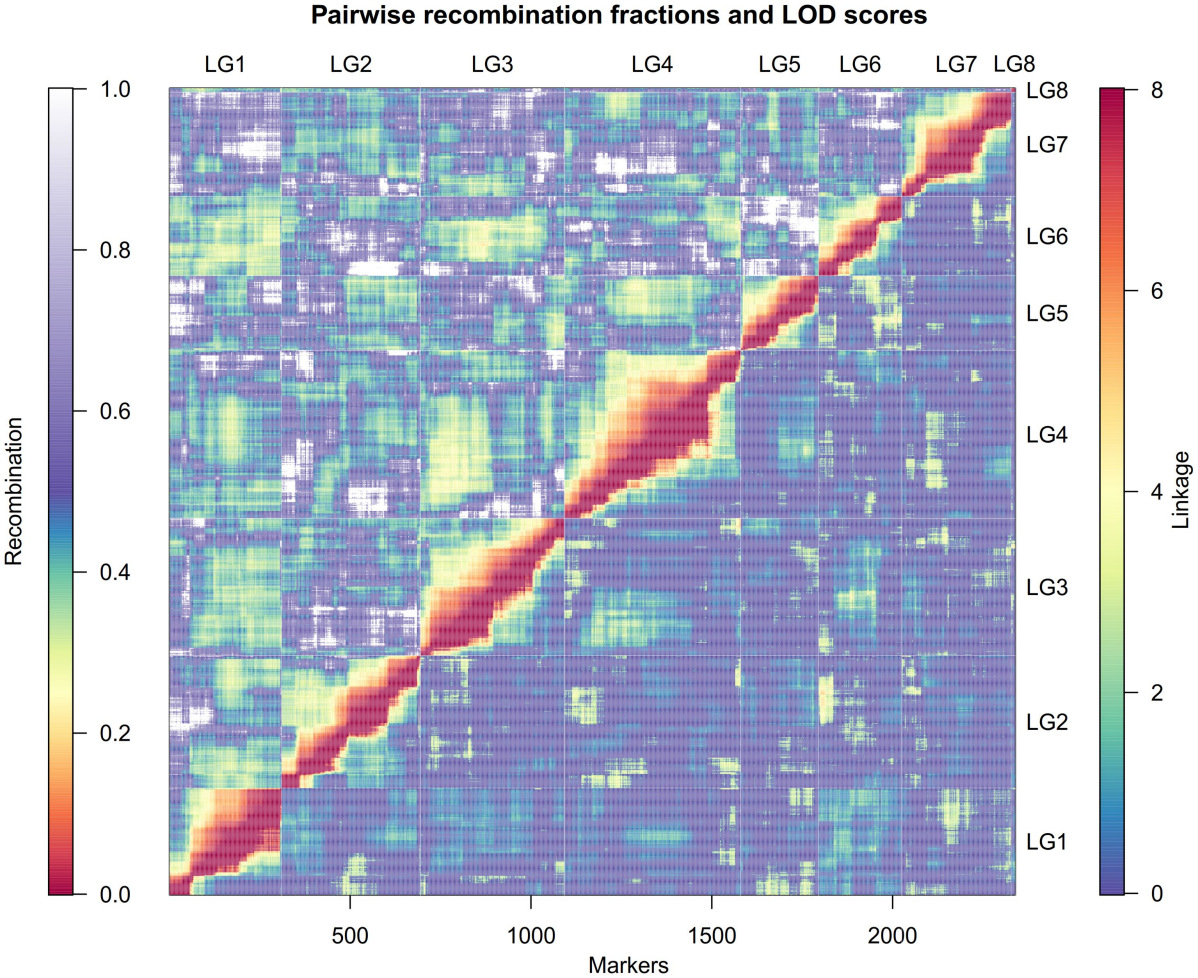
A heatmap demonstrating the estimated recombination fractions and corresponding linkage between the markers in each of the linkage groups. The upper half of the matrix (left and above the diagonal) represent the recombination fraction, with color scale from red (lowest recombination) to purple-white (highest recombination). The bottom half of the matrix (right and below the diagonal) represent the linkage (in LOD score) between each marker pair, with color scale from blue (lowest linkage) to red (highest linkage). The x-scale shows the number of markers.

Additional filtration of the markers and genotypes for segregation distortion, double crossovers and missing data resulted in 2,363 markers and 131 RIL genotypes that were used to construct the linkage map (Supplementary Table 1). The linkage map spread across eight linkage groups (LGs) and spanned a total distance of 545.4cM with a mean marker–marker distance of 0.27cM. The number of markers within LGs ranged from 12 (LG8) to 487 (LG4), and the genetic distance varied from 6cM in LG8 to 94cM in LG2. Mean marker density of the longest LG2 and smallest LG8 was 0.24cM and 0.5cM, respectively (Table 1). Among the eight LGs, LG8 had the highest marker density (3/cM) and LG5 had lowest marker density (2.31/cM; Table 1). The high quality and reliability of the map was confirmed by the recombination fraction and linkage between the markers, as demonstrated in Figure 2. Overall, four significant gaps were observed in the map, ranging from 6cM to 11.4cM in length (the latter found in LG2 between 51.7005cM and 63.0689cM). All of the marker loci of the linkage map matched with the seven pseudomolecules and few unmapped contigs on the lentil genome v1.2 (Supplementary Table 2). There were few mismatches in the orientation of linkage groups with the corresponding pseudomolecule of the lentil genome v1.2 (Table 2). Linkage group LG8 matched regions of pseudomolecule LcChr1 and is proposed to be a portion of LG1 that corresponded to regions of LcChr1.

**Table 1:** Marker distribution over the linkage groups of the linkage map derived from a cross between ILWL 180 and ILL 6002.

Linkage group	Mean distance between adjacent markers (cM)	Mean marker density	Length of linkage group (cM)	Number of mapped markers
LG1	0.20	2.75	63.07	314
LG2	0.25	2.48	94.14	384
LG3	0.21	2.44	83.53	397
LG4	0.19	2.78	91.81	487
LG5	0.29	2.31	63.98	217
LG6	0.27	2.46	68.20	251
LG7	0.25	2.51	74.71	301
LG8	0.50	3.00	5.96	12
Total	0.27	2.59	545.40	2,363

**Table 2:** Distribution of SNP markers between linkage map derived from a cross between ILWL 180 and ILL 6002 and lentil genome v1.2.

Chromosome/Pseudomolecule of lentil genome v1.2	Linkage groups								Total number of markers
	LG1	LG2	LG3	LG4	LG5	LG6	LG7	LG8	
LcChr1	**309**	19	2	12	3	2	0	**12**	359
LcChr2	0	2	**344**	3	0	0	15	0	364
LcChr3	0	**298**	0	0	3	0	0	0	301
LcChr4	0	0	0	**353**	0	0	1	0	354
LcChr5	1	0	0	3	0	**237**	3	0	244
LcChr6	0	0	0	0	**180**	7	2	0	189
LcChr7	0	2	5	2	4	0	**264**	0	277
LcContigs	4	63	46	114	27	5	16	0	275
Total	314	384	397	487	217	251	301	12	**2,363**

*The bold values in each linkage group represent the largest proportion of markers that matched to a single pseudomolecule of lentil genome v1.2*.

### QTL Detection

**Figure 3:**
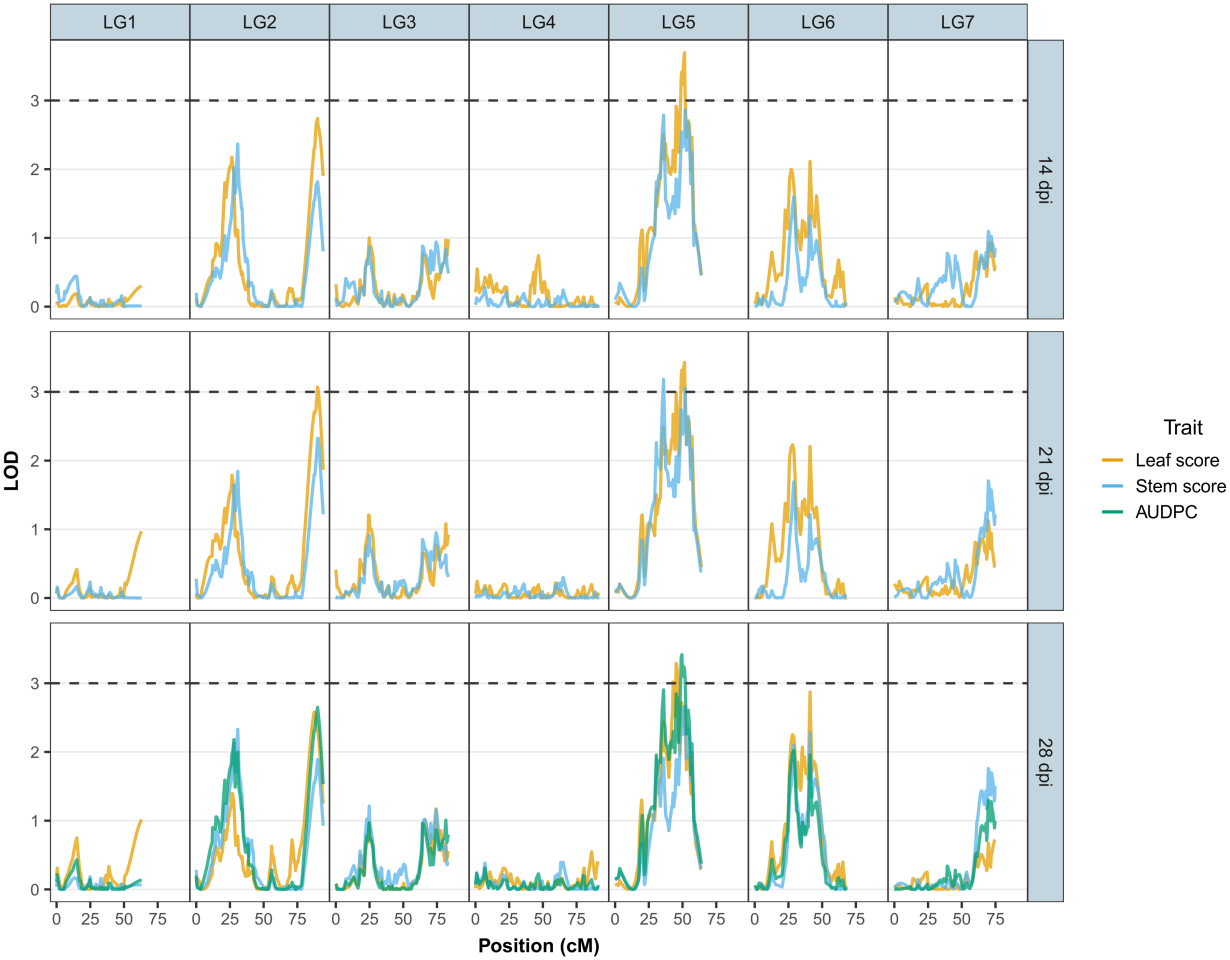
LOD scores for Ascochyta blight disease symptoms (leaf lesions – yellow line and stem lesions – blue line) at 14, 21 and 28days post-inoculation (dpi) and AUDPC (green line) at 28 dpi across the major linkage groups. Linkage group 8 is not presented due to its small number of markers with low LOD scores.

**Figure 4:**
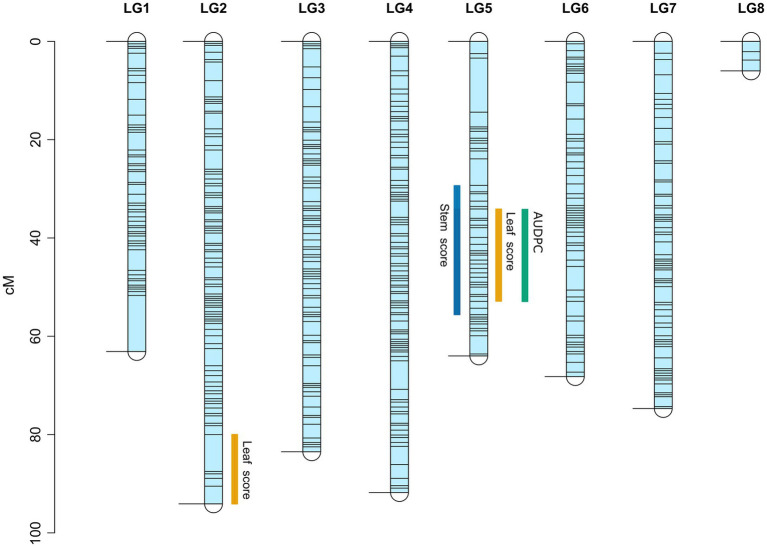
Linkage map of the interspecific RIL population derived from a cross between ILWL 180 and ILL 6002. Black horizontal lines represent the position of genetic markers. Regions of identified QTL for Ascochyta blight disease symptoms are marked with blue, orange and green bars for stem lesions, leaf lesions and AUDPC, respectively.

No-significant differences were detected for AB resistance within 7 dpi among the RIL population and parents; hence, leaf lesion and stem lesion scores at 14, 21 and 28 dpi were considered for QTL analysis. The LOD thresholds determined by R/qtl2 for each trait and at each time point averaged at 2.8; therefore, a more stringent LOD threshold was set at 3.0. Seven QTLs with a significant LOD score of 3 and above were subsequently detected on LG2 and LG5 (Table 3). Of the seven, four QTLs (three on LG5 and one on LG2) were associated with leaf lesion score (yellow graph in Figure 3) while two QTLs on LG5 are linked to stem lesion score (blue graph in Figure 3) and one QTL on LG5 was linked to AUDPC (green graph in Figure 3). Four QTLs linked to leaf lesion score at 14 and 21 dpi, stem lesion score at 21 dpi and AUDPC on LG5 shared a common QTL peak at 51cM and hence are considered as similar and single QTL. Considering this and the remaining three QTLs associated with traits leaf lesion score at 28 dpi and stem lesion score at 21 dpi on LG5 and leaf lesion score at 21 dpi on LG2, four separate QTLs were detected (Figure 4). The phenotypic variance contributed by these QTLs ranged from 9.5 to 11.5 percent (%). BLASTn similarity search of transcripts within the regions of the QTLs on LG5 to the lentil genome assembly v1.2 revealed matches on pseudomolecule LcChr6 and some unanchored contigs. Furthermore, the markers underlying QTL on LG2 matched to genomic regions on pseudomolecules LcChr3 and LcChr1 of lentil genome assembly v1.2.

**Table 3:** Quantitative trait loci for AB resistance determined in the ILWL 180 × ILL 6002 RIL population.

Trait	Days post-inoculation (dpi)	Linkage group (LG)	Position of the QTL peak (cM)	Log of odds (LOD)	Confidence interval (cM)		Phenotype variance (%)
					Low	High	
Leaf lesion score	21	LG2	90.00000	3.07	79.9588	94.1418	9.6
Stem lesion score	21	LG5	35.96569	3.18	29.3309	55.6197	9.9
Stem lesion score	21	LG5	51.50091	3.04	31.0480	57.5027	9.5
Leaf lesion score	28	LG5	45.30773	3.29	34.0957	52.8909	10.3
Leaf lesion score	21	LG5	51.50091	3.43	34.0957	55.6197	10.7
Leaf lesion score	14	LG5	51.50091	3.70	44.4529	55.6197	11.5
AUDPC	–	LG5	51.50091	3.44	34.0957	55.6197	10.7

### Identification of Candidate Genes and Associated Mutations

A survey for QTL-linked markers revealed 118 SNPs underlying the regions within the intervals of identified QTLs. BLASTx similarity search for the annotations of the transcript sequences underpinning the QTL region returned percent identities ranging between 45.42 and 99.10 percent. Among them, the top five annotations for each transcript were filtered based on the highest percent identity and bit score. One best matching and fully characterized putative candidate gene was selected from the top five annotations for each of the transcript (Supplementary Table 3). In total, the leaf, stem lesion score and AUDPC QTLs contained 44 genes.

Analysis of the SNP locations relative to the CDS of each of the 44 candidate transcripts found 20 positioned on the CDS and the rest in the UTR of putative genes (Supplementary Table 4). A comparison of the SNPs located on the CDS with the reference protein sequence of the candidate genes revealed six non-synonymous and 14 synonymous mutations. The putative candidate genes identified with non-synonymous mutations were cinnamoyl-CoA reductase 1; root border cell-specific protein; phenylalanine-tRNA ligase, chloroplastic/mitochondrial; zinc finger CCCH domain-containing protein ZFN-like isoform X2; ferredoxin-dependent glutamate synthase, chloroplastic isoform X1; R3H domain-containing protein 2; and 50S ribosomal protein 5, chloroplastic. The effects of the non-synonymous mutations revealed substitution of an amino acid at specific positions on the coding sequence of these putative candidate genes, as follows: Asparagine (N) 285 Lysine (K); Valine (V) 132 Methionine (M); Isoleucine (I) 155 Valine (V); Leucine (L) 38 Serine (S); Alanine (A) 980 Threonine (T); Leucine (L) 118 Phenylalanine (F); and Alanine (A) 67 Glycine (G; Supplementary Table 4). SIFT analysis predicted that all of the amino acid substitutions are tolerated (>0.05) and no immediate alterations within the corresponding protein structure and associated function are produced.

## Discussion

Interspecific crosses for the introgression of novel alleles from wild to cultivated germplasm are practised to address the genetic bottlenecks that have occurred through recurrent selective breeding processes (Gupta and Sharma, 2007; Tullu et al., 2010; Bhadauria et al., 2017; Dadu et al., 2017). Selection of key traits may then be fast tracked through identification of trait-linked markers and marker-assisted breeding (Bhadauria et al., 2017). In this study, we detail the development of a RIL population derived from *L. culinaris* × *L. orientalis* through aSSD and genotyped using a GBS-t approach that produced a high percentage (98.5%) of reads mapped to the reference transcriptome of the lentil cultivar Cassab. This led to the discovery of many genome wide SNP markers among the parents and inter subspecific RIL population and enabled the identification of QTLs and potential candidate genes conferring resistance to *A. lentis* infection.

The interspecific linkage map constructed in this study included 2,363 markers across eight LGs, corresponding to the seven chromosomes of the lentil genome v1.2 (LG1 and LG8 mapped to the same pseudomolecule LcChr1). The length of the map was shorter (545.4cM) than many previously published maps in lentil (Eujayl et al., 1998; Rubeena et al., 2003; Duran et al., 2004; Hamwieh et al., 2005; Kahraman and Muehlbauer, 2010; De La Puente et al., 2012; Gupta et al., 2012; Ates et al., 2016; Bhadauria et al., 2017; Ates et al., 2018) but was comparable to other recently published maps constructed using SNP markers from GBS-t (432.8cM; Temel et al., 2015) and DArT (497.1cM; Aldemir et al., 2017).

QTL analysis identified three QTLs co-localizing on LG5. Markers underlying these QTLs were located between regions 132,032,337 and 203,277,345bp on the pseudomolecule LcChr6 of the lentil genome v1.2. The genomic region between 51,406,761 and 210,272,490bp on LcChr6 chromosome was previously reported to be associated with AB resistance (Sudheesh et al., 2016a; Polanco et al., 2019). These results indicate that the larger proportion of the resistance at seedling stage is conditioned by common loci and that may be potentially conserved within Genus *Lens* and further agree with Polanco et al. (2019) that common genes are involved in defense responses to *A. lentis* infection. The additional QTL found on LG2 in this study is, on the other hand, novel. This may explain the previously reported superior resistance of ILWL 180 compared to cultivar Indianhead (Dadu et al., 2018b). Benefitted from the availability of largely characterized transcriptome and genome, GBS-t revealed several potential candidate genes with known function underlying the QTLs.

The broader biological processes that these candidate genes are part of included signal transduction, oxidation–reduction process, protein metabolic process, proteolysis, cellular response to stimulus and metabolic processes (Supplementary Table 4). Interestingly, several of the genes identified are directly related to defense responses, such as signal transduction, oxidation–reduction process and autophagy. These results agreed with the previous histological assessments, which showed over-expression of reactive oxygen species (ROS) in the cells of ILWL 180 as a predominant host defense mechanism towards AB infection (Dadu et al., 2018a). Although temporally differentially expressed, similar defense responses in general were also found to operate in the AB resistant lentil cultivars ILL 7537, 964a-46 and CDC Robin against *A. lentis* infection (Mustafa et al., 2009; Khorramdelazad et al., 2018; Sari et al., 2018).

Since three QTLs shared common regions on LG5, there were 24 genes that were identified to be potentially commonly involved among traits including leaf lesion score at 14, 21 and 28 dpi, stem lesion score at 21 dpi and AUDPC. Candidate genes, such as receptor-like protein kinase feronia, zinc finger CCCH domain-containing protein ZFN-like isoform X2 and ubiquitin-conjugation enzyme involved in signal transduction and protein methylene blue sensitivity 1 and thioredoxin F-type, chloroplastic involved in oxidation–reduction process, are found across three time points in both leaflets and stem. The remaining genes were detected to be specific to a trait/s. For example: genes, such as WPP domain-interacting protein 1 and cinnamoyl-CoA reductase 1-like, involved in nucleus organization and cell wall thickening process, respectively, were in play only during 21 dpi in leaflets, whereas genes including cell division cycle protein 48 homologue and triosephosphate isomerase chloroplastic involved in cell division and chloroplast organization were found specific to stem response in resistance to AB (Supplementary Table 4).

A further analysis of potential protein coding effects of SNPs revealed six non-synonymous mutations causing a single amino acid substitution in the predicted protein sequence of the putative candidate genes. Among the six genes with non-synonymous mutations identified in this study, three genes, namely, cinnamoyl-CoA reductase 1-like (Lauvergeat et al., 2001), zinc finger CCCH domain-containing protein ZFN-like isoform X2 (Ciftci-Yilmaz and Mittler, 2008) and ferredoxin-dependent glutamate synthase-chloroplastic isoform X1 (Seifi et al., 2013) are functionally related to plant defense system against pathogen infection. Non-synonymous mutations could potentially alter the structure and function of the corresponding protein and thereby determine host resistance to disease. A non-synonymous SNP causing an amino acid substitution from single glycine to an arginine residue resulted in the drastic modification of the 3D protein structure of an effector *AvrLm4-7* secreted by *Leptosphaeria maculans* (causal agent of stem canker disease in *Brassica napus*) and resulted in the loss of recognition specificity by two resistance genes (*Rlm4 and Rlm7*; Blondeau et al., 2015). Although the SIFT predictive analysis indicated that none of the non-synonymous mutations affect the structure or function of corresponding proteins, these mutations may mediate differential transcript processing and expression to resist *A. lentis* infection. In a similar manner, mutations in non-coding regions, which were not discussed here, may be associated with differential gene expression through modifications of transcription factors binding sites and other epigenetic effects. These non-synonymous and non-coding mutations can be further investigated using predictive bioinformatics models and functional assays, such as targeted perturbation by CRISPR (Lai et al., 2019). Once validated as functional, these markers can be considered as candidate sites for future laboratory induced mutation studies to develop mutant lines with increased resistance to AB.

In all the SNPs within the QTL region, ILL 6002 demonstrated a reference-allele homozygote genotype (0/0) similar to the Cassab reference, while in ILWL 180, all the genotypes were alternative-allele homozygotes (1/1). Though the origin of mutations might be from either parents, given the similarity between the Cassab reference and ILL 6002 in the QTL region and the resistance of ILWL 180, it can be suggested that the SNP mutations have been induced throughout the domestication process of *L. culinaris* and thus may have weakened the defense response system against *A. lentis* found in wild species.

## Conclusion

In conclusion, the present study has utilized a novel interspecific-derived RIL population and reports an interspecific linkage map which will assist in further trait-dissection studies of accession ILWL 180. QTLs identified on LG5 corresponding to pseudomolecule LcChr6 confirm that wild relatives *L. orientalis*, *L. odemensis* and cultivated species *L. culinaris* share a common region associated to AB resistance. The lone QTL on LG2 was not found in earlier studies and can be considered a candidate for gene pyramiding with other novel resistance alleles to produce a diverse gene set for resistance to *A. lentis* infection. To our knowledge, this is the first report of non-synonymous mutations and corresponding amino acid substitutions being identified in lentil in association with disease response to AB. However, an in-depth study is required to reveal the structural and functional changes of the prospective proteins and subsequently propose the molecular mechanisms contributing to associated potential AB resistance in ILWL 180.

## Data Availability Statement

The datasets presented in this study can be found in online repositories. The names of the repository/repositories and accession number(s) can be found at https://www.ncbi.nlm.nih.gov/geo/query/acc.cgi?acc=GSE176412, GSE176412.

## Author Contributions

RD conducted the bioassays, contributed to the data analysis, map construction and QTL analysis and wrote the manuscript. IB analyzed the phenotype data, performed the map construction, QTL analysis, identification of candidate genes and associated mutations and contributed in drafting the manuscript. JC and FR performed assisted single-seed descent method and assisted in drafting the manuscript. SK and SS performed the genotyping of mapping populations and assisted in drafting the manuscript. DG, RF and PS conceived the study, participated in its design and assisted to draft the manuscript. All authors read and approved the final manuscript.

## Conflict of Interest

The authors declare that the research was conducted in the absence of any commercial or financial relationships that could be construed as a potential conflict of interest.

## Publisher’s Note

All claims expressed in this article are solely those of the authors and do not necessarily represent those of their affiliated organizations, or those of the publisher, the editors and the reviewers. Any product that may be evaluated in this article, or claim that may be made by its manufacturer, is not guaranteed or endorsed by the publisher.
